# Route to Cost-Effective Fabrication of Wafer-Scale Nanostructure through Self-Priming Nanoimprint

**DOI:** 10.3390/mi12020121

**Published:** 2021-01-24

**Authors:** Yue Su, Zhaoxin Geng, Weihao Fang, Xiaoqing Lv, Shicai Wang, Zhengtai Ma, Weihua Pei

**Affiliations:** 1 State Key Laboratory of Integrated Optoelectronics, Institute of Semiconductors, Chinese Academy of Sciences, Beijing 100083, China; suyue@semi.ac.cn (Y.S.); fwh@semi.ac.cn (W.F.); lvxiaoqing284@semi.ac.cn (X.L.); mazhengtai@semi.ac.cn (Z.M.); peiwh@semi.ac.cn (W.P.); 2 College of Materials Science and Opto-Electronic Technology, University of Chinese Academy of Sciences, Beijing 100049, China; 3 School of Information Engineering, Minzu University of China, Beijing 100081, China; 4 State Key Laboratory of Crystal Materials, Shandong University, Jinan 250100, China; sc.wang.blikey@mail.sdu.edu.cn

**Keywords:** double-layer, nanoimprint, wafer-scale, nanostructure

## Abstract

Nanoimprint technology is powerful for fabricating nanostructures in a large area. However, expensive equipment, high cost, and complex process conditions hinder the application of nano-imprinting technology. Therefore, double-layer self-priming nanoimprint technology was proposed to fabricate ordered metal nanostructures uniformly on 4-inch soft and hard substrates without the aid of expensive instruments. Different nanostructure (gratings, nanoholes and nanoparticles) and different materials (metal and MoS_2_) were patterned, which shows wide application of double-layer self-priming nanoimprint technology. Moreover, by a double-layer system, the width and the height of metal can be adjusted through the photoresist thickness and developing condition, which provide a programmable way to fabricate different nanostructures using a single mold. The double-layer self-priming nanoimprint method can be applied in poor condition without equipment and be programmable in nanostructure parameters using a single mold, which reduces the cost of instruments and molds.

## 1. Introduction

Ordered metal nanostructures with the plasmonic effect have important applications in various fields, such as localized surface plasmon resonance, surface-enhanced Raman scattering (SERS), and energy conversion and storage [1,2,3,4]. The conventional techniques used to fabricate ordered nanostructures primarily focus on electron beam lithography and focused ion beam lithography (FIB) [5,6,7]. However, these techniques are extremely time consuming and expensive when used to fabricate nanostructures in a large area. Alternatively, non-traditional lithography technologies for large-area patterning were developed, including nanoimprint lithography and soft lithography [8]. For nanoimprint lithography, the mold is hard, expensive, and easily broken, owing to the high pressure applied to it, typically of the order of 10^9^ Pa [9]. For soft lithography, the mold is typically made of elastomer, including polydimethylsiloxane (PDMS), which is used extensively [10]. Soft lithography involves microcontact printing [11], solvent-assisted nanoscale embossing [12], and soft interference lithography [13]. Capillary force lithography combines the essential feature of nanoimprint lithography (a polymer melt) with that of soft lithography (the use of a soft mold) [8]. The polymer layer can fill the PDMS mold automatically through capillary force by raising the temperature above the glass transition temperature (*T_g_*) after solvent evaporation (temperature-induced capillarity) or by molding prior to solvent evaporation (solvent-induced capillarity) at room temperature [14,15,16]. In the imprint process using capillarity, no pressure is applied, thereby removing the need for an expensive state-of-the-art imprinter to provide a high temperature and pressure, which has potential for mass industrialization with low cost.

The imprinted pattern is typically formed with an overcut profile, owing to the facile mold release, which causes lift-off difficulty. One method to transfer the imprinted nanostructure to a functional material is to dry etch the residual of the imprinted resist and the functional material under it using an expensive inductively coupled plasma (ICP) system [17,18,19]. The expensive instrument limits the transferring of the functional material, both in cost and by imposing poor conditions. Another method, which is significantly cheaper, is to apply an additional lift-off layer (LOL) to the substrate before it is coated with the imprint resist [20,21,22,23]. Because the developing rate of the LOL is faster than that of the imprint resists, the developing LOL can penetrate beneath the imprint resist pattern to form an undercut profile favorable to lifting off metal in a chemical solution. Furthermore, the thickness of transferring functional material is restricted by the thickness of the LOL, instead of the thickness of the nanoimprint mold, which provides a programmable route to adjust the thickness of the functional material using a single stamp.

Most of the nanoimprint methods are capable of generating a perfect negative replica of the original stamp. Therefore, if different features are desired, new molds are necessary to create every new pattern, which increase the cost of molds. To reduce the dependence of nanoimprint on primary mold, there are some innovative approaches to generating patterns that are not a mere negative replica of the original master, such as stress relaxation in a elastomer master [24], swelling and deswelling of the stamp during patterning [12], and combining nanostructure mold with flat plate [25]. All these methods to control the parameters of nanostructure are based on changing of molds. Here we provide a new way to control the nanostructure parameter by changing the thickness of imprint resist and the developing condition, instead of changing the mold. 

Here we report a cost-effective double-layer self-priming nanoimprint method that can generate metal gratings with programmable width and height from PDMS mold replicated from digital video disk (DVD). The width can be varied from 200 nm to 350 nm by increase the thickness of top photoresist and reduced subtly by increasing the developing time. The height can be adjusted by the thickness of bottom photoresist with largest value of 230 nm which is larger than the height of original mold. This method has been applied to fabricate gold gratings on hard and soft substrate uniformly in 4-inch scale with low cost. Besides, the nanostructure was extended to silicon nanodot arrays by imprinting twice with a PDMS mold, replicated from a DVD, combining with etching. With the PDMS mold replicated from the silicon nanoparticles, gold nanoparticles and nanoholes were fabricated using double-layer self-priming nanoimprinting with a different developing time. It’s another proof of versatility of double-layer system in changing nanostructure parameters by developing. Moreover, this cheap nanoimprinting method combined with dry etching can also be applied to pattern 2D material gratings. This proves that the double-layer self-priming nanoimprinting method can be widely applied to pattern different materials. In conclusion, the nanostructure can be adjusted using a single mold without expensive instruments, which reduce the cost of instruments and molds. Different nanostructure of different material was patterned on soft and hard substrate, which illustrates the wide application of the double-layer self-priming nanoimprint.

## 2. Materials and Methods 

### 2.1. PDMS Mold Fabrication

Commercially available DVD consists of two polycarbonates (PC) surfaces that sandwich a reflective layer and a dye layer between them. The two PC layers were manually separated by splitting DVD along the edge. The transparent PC side with gratings was cleaned ultrasonically with ethyl alcohol and deionized water to remove the dye layer. The PDMS was prepared by mixing 20 g of pre-polymer (Sylgard 184 from Dow Corning) with a curing agent in a 10:1 ratio at room temperature. The mixture was then poured on DVD templates, degassed to remove air bubbles, and cured at 70 °C for 2 h. After that, the PDMS stamp formed with negative patterns of the DVD was manually peeled off. The color strip could be observed on PDMS indicating that the 1D gratings pattern on PC was transferred to PDMS. The morphology of DVD and PDMS stamps were characterized by an atomic force microscope (AFM, Digital Instrument, Santa Barbara, CA, USA) shown in Figure S1a,b, respectively. The height of gratings on DVD and PC was 200 nm and 140 nm, as shown in Figure S1c. The relief height was lower in PDMS due to the low modulus of PDMS [26]. This could be addressed by h-PDMS/PDMS composite stamp; however, the process was very complicated [11]. For simplification, we still chose PDMS as a template; because the 1D grating pattern was transferred successfully to PDMS from DVD, although it was deformed comparing with an original stamp. 

### 2.2. Double-Layer Self-Priming Lithography on Hard and Soft Substrates 

**Scheme 1:**
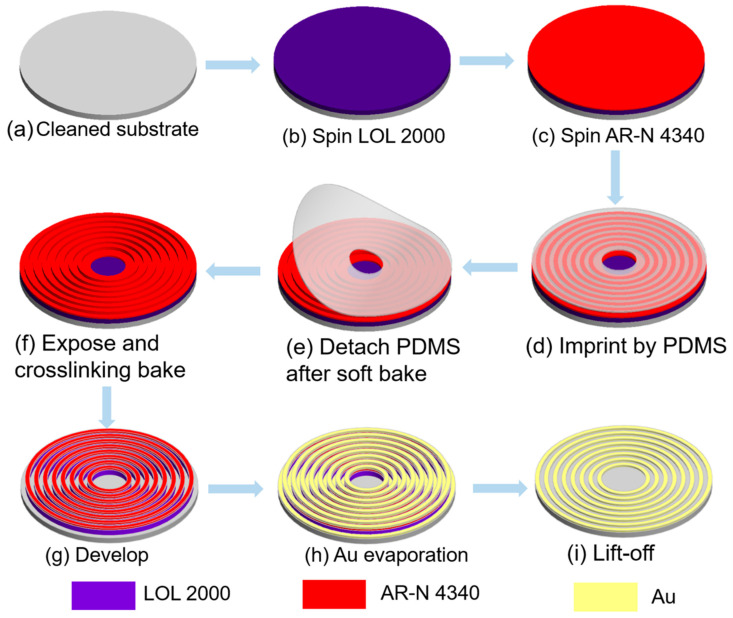
Schematic of the fabrication processes of gold gratings by double-layer self-priming nanoimprinting on hard substrates.

The process of the double-layer self-priming nanoimprint on hard substrate (silicon and quartz) was illustrated in Scheme 1. First, the silicon and quartz wafers were cleaned using piranha (the volume ratio of H_2_SO_4_ to H_2_O_2_ is 3:1) heated on a hot plate at 90 °C for 30 min, rinsed thoroughly using deionized water and dried in a stream of nitrogen. The cleaned substrates were coated with Hexamethyldisiloxane layer by vapour priming to promote adhesion of photoresist. Next, bottom photoresist LOL 2000 (Shipley Company, Marlborough, MA, USA) was spin-coated at speed of 6000 revolutions per minute (rpm) on the prepared substrate and cured on hot-plate at 150 °C for 3 min (Scheme 1b). Subsequently, the substrate with cured LOL was spin-coated with top photoresist AR-N 4340 (Allresist, GmbH, Strausberg, Germany) at a speed of 6000 rpm which was diluted to 1:4 with AR 300-12 (Allresist, GmbH, Strausberg, Germany) so that the thickness of the photoresist could be decreased to 100 nm (Scheme 1c). The PDMS stamp with gratings relief was gently placed on the photoresist to avoid air bubbles (Scheme 1d). After standing for several minutes (the time is not important parameter as discussed in Section 3.4 and Figure S6), the liquid AR-N 4340 automatically filled relief of PDMS stamp by capillary force; therefore, the color strip caused by diffraction was disappeared. Then, the substrate covered with PDMS was heated at 110 °C on the hot plate for 150 s to soft-bake the AR-N 4340; this was followed by detaching the PDMS mold from the substrate (Scheme 1e). The colour strip was observed on the top photoresist proving that the nanostructure on the PDMS stamp was transferred to the top photoresist. Subsequently, the photoresist was overexposed at 220 mJ/cm^2^ at 365 nm and crosslinking-baked at 120 °C on the hot plate for 120 s (Scheme 1f). If the top photoresist is positive, the process of exposure and crosslinking-bake is unnecessary so that this method can be applied in the laboratory even without a lithography machine. Afterwards, the photoresist was developed with the concentration of Tetramethylammonium hydroxide (TMAH, Aladdin, Shanghai, China) 1.2% diluted in water at a total volume of 300 mL and rinsed in deionized water twice for 10 s (Scheme 1g). The developing process was depicted by the scanning electron microscope (SEM) image of photoresist with different developing times in Figure S2. The residual of the top photoresist was dissolved, then the LOL was exposed and dissolved quickly to penetrate under the top photoresist. Consequently, the undercut was formed. The developing time and concentration were optimized to ensure the substrate was exposed. The structures of the photoresist after developing were observed by scanning electron microscope (SEM, HITACHI SU8020 field emission scanning electron microscope, Hitachi, Tokyo, Japan). Finally, 2 nm Cr and 100 nm Au was deposited (deposition rate of 0.1 nm/s) onto the substrate patterned with gratings by electron-beam deposition (Scheme 1h). Then, it was immersed in the N-Methyl pyrrolidone (NMP, Suzhou Research Semiconductor Co., Ltd., Suzhou, China) to remove the photoresist; this process is called lift-off (Scheme 1i). The experimental conditions for subsequent parts of the study are the same as above, unless otherwise stated.

For soft substrates (polyimide (PI) and Parylene-C film), they were attached to silicon wafer before conducting the double-layer self-priming nanoimprint. The polyimide (PI-2611, DuPont, Wilmington, DE, USA) was spin-coated on a cleaned silicon wafer followed by a curing process [27]. The parylene-C, with a thickness of 10 μm, was deposited on a silicon wafer using chemical vapor deposition (CVD) [28]. After metal evaporation and lift-off, the soft substrates were detached from silicon wafer easily by immersing in water to get the flexible film with the gold nanostructure. The schematic diagram of the two steps for soft substrates was supplied in Figure S3.

### 2.3. Gold Nanoparticle and Nanohole Arrays Fabrication 

**Scheme 2:**
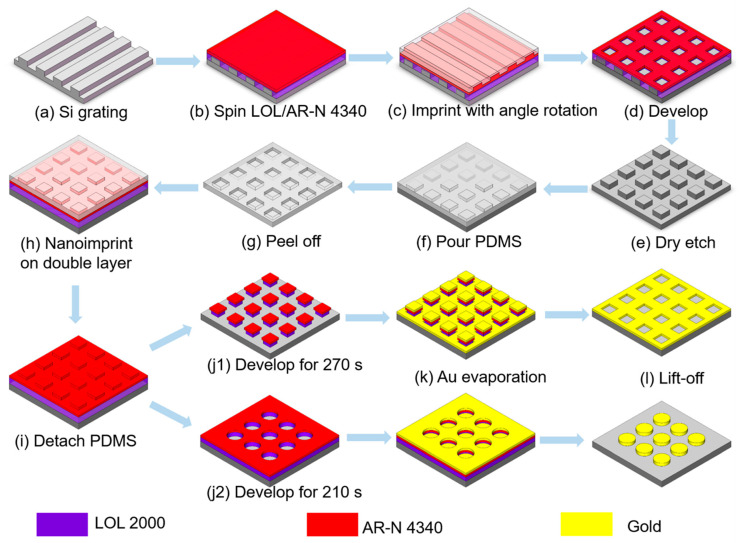
Schematic of the fabrication processes of gold nanohole and nanoparticle arrays using double-layer self-priming nanoimprinting.

The 1D grating mold was applied to fabricate silicon nanoparticle arrays by combining self-priming imprint with dry etching twice. Using PDMS mold replicated from the silicon nanoparticle arrays, gold nanoparticles and nanoholes were fabricated by the double-layer self-priming imprint. The specific process was shown in Scheme 2. The silicon wafer with 1D grating photoresist after developing was etched by SF_6_/BCl_3_/Ar_2_ for 150 s (Plasmalab System 100, Oxford Instruments, Abingdon, UK) and the residual photoresist was dissolved by NMP (Scheme 2a). The silicon grating chip was coated by a LOL 2000/AR-N 4340 double layer system as described above (Scheme 2b). Next, the liquid AR-N 4340 was nanoimprinted by the PDMS stamp replicated from DVD with rotation angle to the silicon gratings (Scheme 2c). After developing by TMAH (Scheme 2d), the exposed silicon was etched by SF_6_/BCl_3_/Ar_2_ for 150 s so that the nanoparticle arrays were fabricated on the silicon substrate (Scheme 2e). After removing the residual photoresist by NMP, the silicon nanoparticle arrays were coated with Trichloro(1*H*,1*H*,2*H*,2*H*-perfluorooctyl) silane (Sigma-Aldrich, St. Louis, MO, USA) by chemical deposition in a vacuum chamber for 12 h. Subsequently, PDMS was poured on the silicon template and cured (Scheme 2f). After detaching the PDMS from the silicon template, the PDMS stamp was formed with negative patterns of the silicon nanoparticle (Scheme 2g). The morphology of silicon and PDMS stamps were characterized using AFM, as shown in Figure S4. Then, the silicon substrate with a size of 2 cm × 2 cm was nanoimprinted with the nanohole PDMS stamp by a double-layer self-priming nanoimprint method (Scheme 2h). In the developing process, the photoresist in the center of the raised nanoparticles, was dissolved first, and the photoresist on the raised nanoparticles was dissolved last. Thus, the formation of the nanoholes of the photoresist required a shorter developing time (Scheme 2(j2)), while the nanoparticles of the photoresist required a longer developing time (Scheme 2(j1)). After the gold deposition (Scheme 2k) and lift-off (Scheme 2l), gold nanoparticle and nanohole arrays were fabricated inversely to the photoresist patterns.

### 2.4. MoS_2_ Gratings Fabrication

A single layer of MoS_2_ sheet was grown on a sapphire substrate using CVD. Polymethyl methacrylate (PMMA) was spin-coated on a MoS_2_ sheet and cured at 180 °C on a hot plate for 15 min. The MoS_2_ sheet coated with PMMA was released from the sapphire substrate in an NaOH solution. It was then carefully transferred to an SiO_2_ substrate in deionized water so that the MoS_2_ sheet adhered to the SiO_2_ substrate. The PMMA was dissolved by acetone, and the MoS_2_ sheet on the SiO_2_ substrate was then cleaned using ethyl alcohol and deionized water; it was then cured at 120 °C on a hot plate for 10 min. The newly prepared MoS_2_ sheet was characterized by Raman spectra. Subsequently, the double-layer self-priming nanoimprint method was conducted on the SiO_2_ substrate coated with a MoS_2_ sheet, as shown in Scheme 1. After developing, the photoresist grating was transferred to the MoS_2_ by etching the exposed MoS_2_ using oxygen plasma at 100 W with an oxygen flow rate of 1 L/min for 4 min (PDC-32G, Harrick Scientific Products, Inc., New York, NY, USA). The photoresist on the MoS_2_ was then dissolved using NMP.

### 2.5. Reflection Spectra Detection 

The reflection spectra of 1D gold gratings on silicon were detected for different incident angles at different points of the 4-inch silicon using an angle-resolved R1-UV series spectroscopy meter (Ideaoptics, Shanghai, China). The polarization direction was perpendicular to the slit of the gratings. The beam size on-chip was 1 mm^2^, and the resolution of the spectrograph was 0.44 nm. To obtain the refractive index sensitivity, the gold gratings on silicon were separated into 4 × 4 mm portions and were immersed in different concentrations of sucrose solution to record the reflection spectra.

### 2.6. Raman Characterizations 

The Raman characterizations of the MoS_2_ nanograting were performed in a LabRAM HR-Evolution Raman microscope (Horiba Jobin Yvon, Kyoto, Japan), equipped with a 532 nm laser and 600 g/mm grating. The laser was focused on a 100× objective, and the power was set to 1 mW to avoid any damage of the sample. The Raman mapping step size was 500 nm.

## 3. Results

### 3.1. Gold Gratings Fabricated on Different Substrates

**Figure 1:**
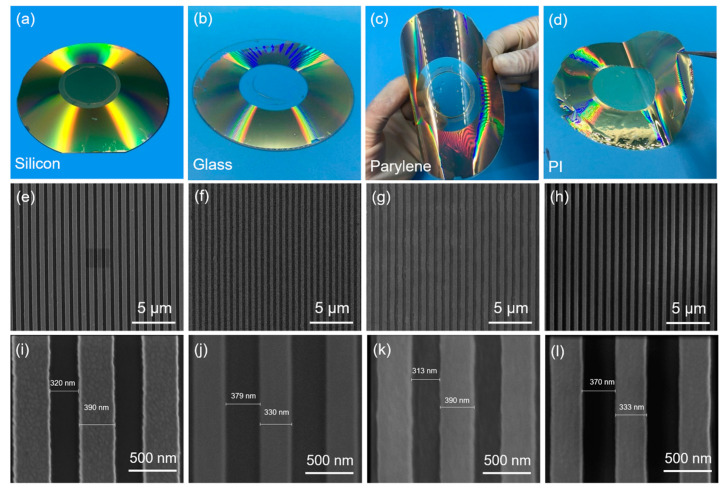
The photographs of gold gratings on different substrates and SEM images with different amplification. The photographs of gold gratings on (**a**) silicon, (**b**) glass, (**c**) parylene-C, and (**d**) PI. SEM images of gold gratings with different amplification on (**e**,**i**) silicon, (**f**,**j**) glass, (**g**,**k**) parylene-C film and (**h**,**l**) PI.

Using the PDMS stamp replicated from cheap DVD, the double-layer self-priming nanoimprint technology was implemented on hard and soft substrates with 4-inch size, including silicon wafer, quartz, Parylene-C, and PI. The spin speed of AR-N 4340 was 3000 rpm and concentration of TMAH was 1.6%; other conditions were the same as those described in Section 2.2. The photographs and SEM images of gold gratings on different substrates are shown in Figure 1. After evaporating the gold with a height of 100 nm, the gold was successfully lifted-off on different substrates. The gold gratings after lift-off were complete on all four substrates, as observed from the photograph in Figure 1a–d. The soft film could be curved at liberty without incurring damage. The gold gratings were uniform and did not exhibit defects in the tens of micrometers scale on all four substrates, as shown in Figure 1e–h. The widths of the gratings were 390, 330, 390, and 333 nm on the silicon wafer (Figure 1i), quartz (Figure 1j), parylene-C (Figure 1k), and PI (Figure 1l), respectively. The error of the width on different substrates was 60 nm. In conclusion, the gold gratings were successfully fabricated on hard and soft substrates in the wafer-scale using double-layer self-priming nanoimprint technology with a PDMS stamp replicated from a DVD. 

Because silicon wafer is conductive and easy to split among four substrates, the nanostructure on silicon could be observed clearly by an SEM. In the following, the uniformity of the nanostructure on a 4-inch silicon wafer was investigated using a SEM and reflection spectra. Subsequently, we discuss two important factors in the morphology of photoresists on silicon: (1) the cross-linking bake temperature of the top photoresist (AR-N-4340), which is critical for forming an undercut; (2) the thickness of the top (AR-N-4340) and bottom (LOL 2000) photoresists, which may adjust the linewidth and the maximum height of the evaporated metal, respectively. The double-layer self-priming is then successfully extended to fabricate other metal nanostructures (nanoparticles and nanoholes) on silicon. Subsequently, we investigate the properties of refractive index sensing with gold gratings on silicon as an example of their application to a plasmonic sensor. Finally, the double-layer self-priming nanoimprint method was also applied to pattern two-dimensional materials like MoS_2_. 

### 3.2. Characterization of Uniformity 

One of the most important characters of nanostructures in a large area is the uniformity. The uniformity was characterized by SEM image and reflection spectra of different points on 4-inch silicon. The two different methods both indicate that the gratings on the 4-inch silicon are uniform, with a difference not exceeding 30 nm.

**Figure 2:**
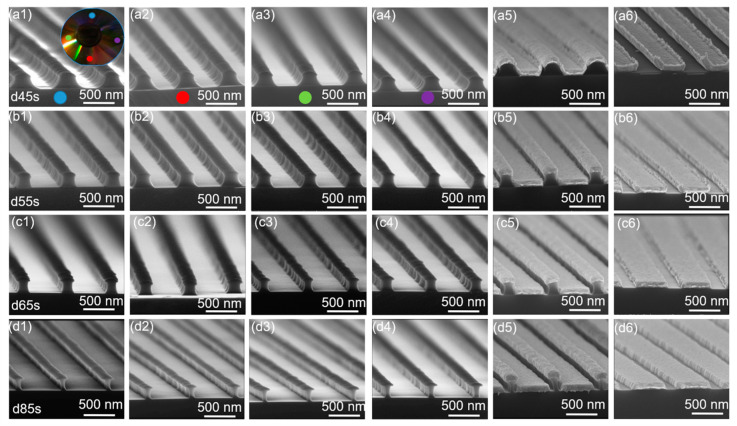
The SEM images of gratings patterned with photoresist under different developing times (**a**–**d**) in different places marked in the inset of (**a1**) on the silicon wafer (**1**–**4**). (**a5**–**d5**) is the SEM image after Au evaporation of different developing time and (**a6**–**d6**) is the SEM image of gold gratings after lift-off.

**Figure 3:**
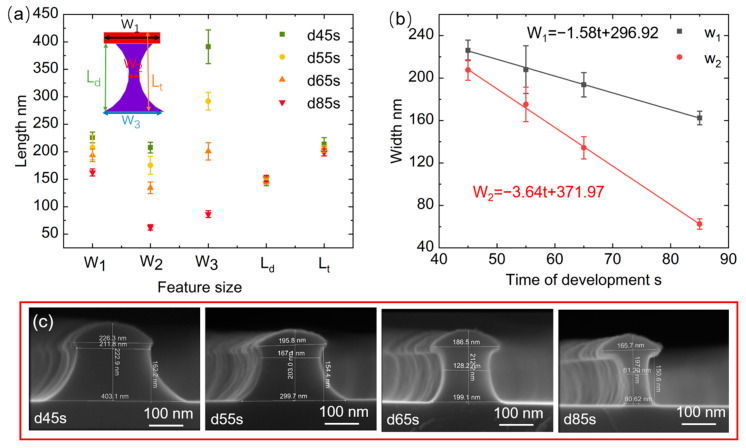
The feature size changing with the developing time. (**a**) The average value and standard deviation of feature size calculated from photoresist gratings under different developing times shown in Figure 2; (**b**) the width of top photoresist presented by *w*_1_ and the width of bottom photoresist presented by *w*_2_ vary with developing time. The linear fitting of the width versus the developing time indicates that the developing speed of the top photoresist is 1.58 nm/s and the developing speed of bottom photoresist is 3.64 nm/s. (**c**) The SEM image of photoresist on silicon developing for different time labelled with size.

The gratings of the photoresist on silicon after developing were measured using an SEM on 4 different points marked by colorful dots in the inset of Figure 2(a1). The four points are far from each other, with a distance larger than 3 cm. The SEM images of the photoresist at the four different points are depicted in Figure 2(1)–(4). The SEM images of gratings developed with 1.2% TMAH for a different developing time are shown in Figure 2a–d. Intuitively, there was minimal difference in the photoresist morphology between the four points at a 4-inch wafer scale, observed from the SEM images. The feature size was measured precisely using profile SEM images labeled with the scale (Figure 3c). The average value and standard deviation were calculated using the length of the feature size (labeled in the inset of Figure 3a measured at four different points on one chip. The width of the gold gratings after lift-off depends on the width of the top photoresist *w*_1_ (labeled in the inset of Figure 3a). The standard deviation of *w*_1_ did not exceed 15 nm, which proves that the gratings were uniform on the wafer-scale.

The sidewall of photoresist can vary from curve to straight with *w*_3_ decreasing rapidly for an increasing developing time. The steep sidewalls of gold gratings can be obtained if the width of the top photoresist *w*_1_ is wider than the largest width of bottom photoresist *w*_3_ as shown in Figure 2d6. To realize a steep sidewall of gold gratings, the developing time should be adjusted carefully to ensure *w*_3_ was smaller than *w*_1_. In Figure 2(a5,a6), although the sidewall was covered with gold, the undercut was still formed and the lift-off was successful. It reveals the large fault tolerance in the experiment condition for lift-off through the double-layer method. 

The widths *w*_1_ and *w*_2_ for different developing times were linear-fitted to obtain the developing rate of the top (1.58 nm/s) and bottom (3.64 nm/s) photoresists (Figure 3b). The developing rate of the bottom photoresist was more than two times faster than that of the top photoresist. By changing the developing time, the linewidth of the top photoresist could be varied for a few tens of nanometers (from 225 to 165 nm). Adjusting the developing time provided a method to subtly modulate the linewidth of the gold gratings. The SEM images of the photoresist gratings at the four points of the 4-inch silicon is uniform, exhibiting a difference of no more than 30 nm.

**Figure 4:**
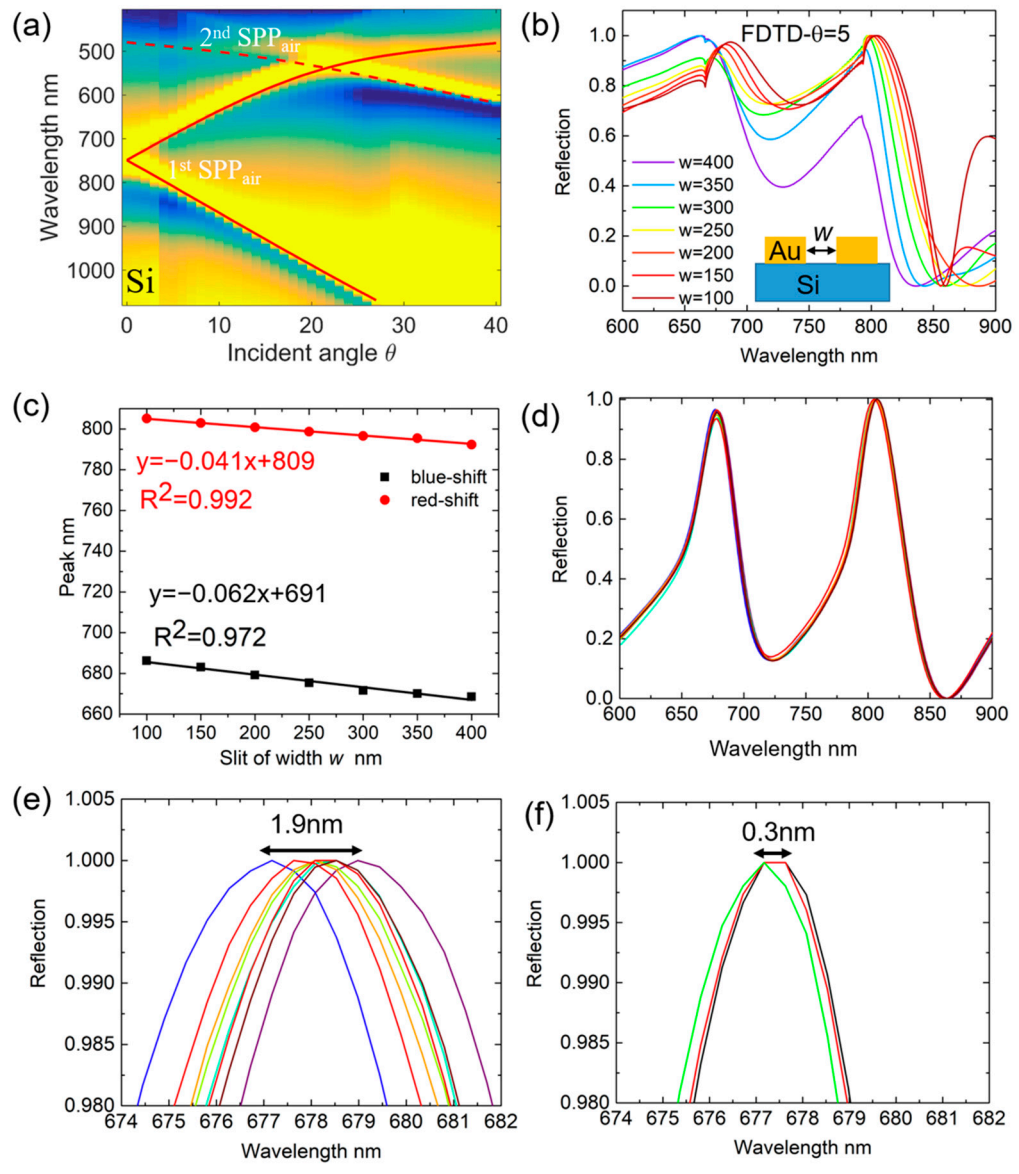
The reflection spectra of gold gratings at different points. (**a**) The reflection spectra on silicon measured with different incident angles. (**b**) The finite-difference time-domain (FDTD) simulation of gold gratings with different width of slit (*w*) on silicon with incident angle of 5°. (**c**) Linear fitting of the position of reflection peak with the width of slit obtained from (**b**). (**d**) The reflection spectra of gold gratings on silicon measured on 8 points on 4-inch silicon wafer. (**e**) The enlarged view of (**e**) at the blue-shift peak. (**f**) The blue-shift peak measured at 3 points on 4 × 4 mm gold gratings on silicon.

Another method to illustrate the uniformity of metal nanostructure is to measure the reflection spectra of gold gratings on silicon which is sensitive to the geometry of gratings. As shown in Figure 4a, the position of the measured reflection peaks were coincident with surface plasmon polaritons (SPPs), presented by red lines, as described in [29]

(1) $$\lambda_{spp}=\frac{p}{m}\left(\sqrt{\frac{\varepsilon_{d}\varepsilon_{m}}{\varepsilon_{d+}\varepsilon_{m}}}\pm sin\theta \right),m=\pm 1,\pm 2,\cdots$$

where:

*ε_m_* and *ε_d_* is the permittivity of the metal and the surrounding media; 

*p* is the period of gold gratings;

*θ* is the incident angle; 

*m* is the order of SPP.

The peaks of first-order SPPs on gold gratings and air were split into two branches as the incident angle increased. Thus, the detection wavelength can be modulated for a different incident angle to match the requirement of the instruments, including the source and spectrograph.

For the reflection detection of different points on the 4-inch silicon wafer in air, at the incident angle of 5° (Figure 4d and the enlarged view Figure 4e), the position of the reflection peaks differs by an error of 1.9 nm in the blue-shift branch. Using a finite-difference time-domain (FDTD) simulation to analyse the cause of the non-coincidence of the reflection peak, the reflection peak was varied with the width of the slit (Figure 4b). Using a linear fit for the peak position as it varies with the width of the slit, the peak position of the blue-shift and red-shift branches can reduce by 6.2 nm and 4.1 nm when the width of the gold gratings slit increases by 100 nm. The 1.9 nm difference in the blue-shift peak position in the 4-inch silicon was caused by a 30 nm difference of the width; this is in good agreement with the results of the SEM images shown in Figure 2 and Figure 3. Although the peak position difference was 1.9 nm on the 4-inch silicon, it can be reduced to 0.3 nm (smaller than the resolution of the spectrometer) on the 4 × 4 mm wafer portions, corresponding to a 5 nm difference in the width of the gold gratings (Figure 4f). The peak position is not affected by the morphology difference in such a small area, which indicates that the gold gratings can be applied to a sensor without the influence of nanostructure differences. The reflection spectra illustrate a 30 nm difference in the width of the gold gratings in the 4-inch area and a 5 nm difference in the 4 × 4 mm area.

### 3.3. Effect of Crosslinking Bake Temperature 

The critical condition to form undercut by the double-layer system is that the developing rate of LOL is larger than that of AR-N 4340. The developing rate of AR-N 4340 can be reduced by improving the temperature of the crosslinking-bake. This conclusion could be easily verified by photolithography on structure with a feature size of micrometre shown in Figure S5. Under the same condition of developing, the exposed AR-N-4340 was thicker when the crosslinking-bake temperature increase. 

**Figure 5:**
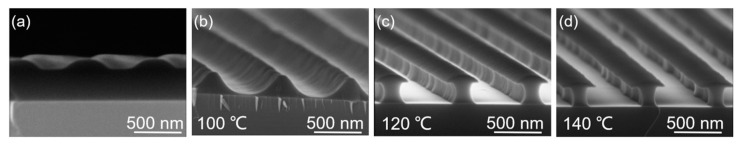
The effect of crosslinking-bake temperature of top negative photoresist AR-N 4340 with the same time of 120 s on the morphology of photoresist developing with 1.6% tetramethylammonium hydroxide (TMAH). (**a**) The SEM image of photoresist before developing. (**b**) The SEM image of photoresist with crosslinking-bake temperature of 100 °C, developing for 10 s. (**c**) The SEM image of photoresist with crosslinking-bake temperature of 120 °C, developing for 40 s. (**d**) The SEM image of photoresist with crosslinking-bake temperature of 140 °C, developing for 80 s.

For this experiment, the spin speed AR-N 4340 was 3000 rpm and the concentration of TMAH was 1.6%; other condition remained the same as described in Section 2.2, except for the varied cross-linking temperature. The developing time was optimized to expose the substrate. When the crosslinking-bake temperature was 100 °C, AR-N 4340 was dissolved quickly; thus, the step failed to form undercut as shown in Figure 5b. For a higher crosslinking bake temperature of 120 °C (Figure 5c) and 140 °C (Figure 5d), the undercut can be successfully formed using developing. When the crosslinking-bake temperature was 140 °C, a longer time was required to dissolve the residual of the AR-N-4340. Thus, the developing time was twice as long as that for the crosslinking-bake temperature of 120 °C to expose the substrate. The linewidth of AR-N-4340 was 320 nm and 370 nm when the crosslinking-bake temperature was 120 °C and 140 °C, respectively. The linewidth of the top photoresist can be wider when the developing rate difference of two photoresists is larger. Here, we considered the crosslinking-bake temperature of 120 °C as the optimal condition because it enables the formation of a good undercut and requires a shorter developing time.

### 3.4. Effect of Photoresist Thickness

**Figure 6:**
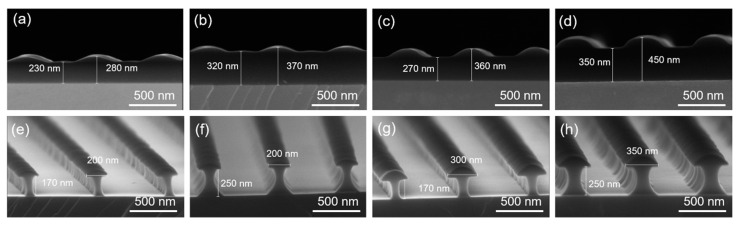
The width of the top photoresist and the thickness of the lift-off layer (LOL) can be adjusted by changing the spin speed of AR-N 4340 and the LOL, respectively. (**a**) The SEM image before developing with both the AR-N 4340 and LOL spin speeds at 6000 rpm. (**e**) The SEM image for (**a**) after developing with 1.2% TMAH for 85 s with a top photoresist width of 200 nm and LOL thickness of 170 nm. (**b**) The SEM image before developing with an AR-N 4340 spin speed of 6000 rpm and LOL spin speed of 2000 rpm. (**f**) The SEM image for (**b**) after developing with 1.2% TMAH for 85 s with a top photoresist width of 200 nm and LOL thickness of 250 nm. (**c**) The SEM image before developing with an AR-N 4340 spin speed of 3000 rpm and LOL spin speed of 6000 rpm. (**g**) The SEM image for (**c**) after developing with 1.6% TMAH for 40 s with a top photoresist width of 300 nm and LOL thickness of 170 nm. (**d**) The SEM image before developing with an AR-N 4340 spin speed of 2000 rpm and LOL spin speed of 2000 rpm. (**h**) The SEM image for (**d**) after developing with 1.6% TMAH for 40 s with a top photoresist width of 350 nm and LOL thickness of 250 nm.

It is a programmable way to adjust the height and the width of gold gratings by changing the thickness of LOL and AR-N 4340, respectively. In this experimental setup, the maximum height of the gold gratings was equal to the thickness of the LOL, which could be adjusted from 170 to 250 nm by changing the spin-coating speed from 6000 to 2000 rpm. Interestingly, the nanoimprint height difference (NHD) could be adjusted by the spin-coating speed of AR-N 4340; thus, the width of the top photoresist *w*_1_ (shown in the inset of Figure 3a) could be modulated. This property offers a method to fabricate nanostructure with different feature size by a single mold. The SEM image of photoresist on the substrate before developing depicted in Figure 6a–d, the nanoimprint height difference was 50 nm, 50 nm, 90 nm, and 100 nm when the spin-speed of AR-N 4340 was 6000 rpm, 6000 rpm, 3000 rpm, and 2000 rpm, respectively. The NHD was smaller than the height of PDMS mold (140 nm). The reduction in the printed feature dimension was owing to the volumetric shrinkage accompanied by removal of solvent through PDMS during imprinting [16,30]. According to previous work [31], the capillary kinetics for a permeable mold and small polymer-film thickness was given by

(2) $$\frac{z}{h_{0}-z}-ln\left(\frac{h_{0}}{h_{0}-z}\right)=\frac{\gamma cos\theta }{6\eta L}$$

where:

*h*_0_ is the primary thickness of polymer;

*z* is the capillary rise in time t (i.e., the NHD);

*L* is the half-channel width;

*γ* is interfacial tension;

*θ* is the contact angle;

and *η* is the viscosity of the polymer melt. 

The AR-N 4340, with the low viscosity of 18 mPa∙s according to the datasheet, fills the PDMS relief quickly without a difference of NHD under a different imprint time of 5 min and 1 h (Figure S6). The right-hand side of Equation (2) does not vary for the same photoresist. By changing the spin speed, the only varied parameter is the primary thickness of AR-N 4340. The NHD increases when the primary thickness of AR-N 4340 becomes larger, which was in good agreement with the experiment result.

The widths of the top photoresist *w*_1_ after developing were 200 nm, 200 nm, 300 nm, and 350 nm, as shown in Figure 6e–h. The width of top photoresist increased with an increase in the NHD. Comparing Figure 6e,f, when the LOL was higher, the linewidth of LOL near the substrate *w*_3_ was wider than the linewidth of top photoresist *w*_1_ (shown in the inset of Figure 3a), which causes the curve sidewall of evaporated metal. The LOL should be thinner if the straight sidewall was required. Thus, the maximum thickness of evaporated metal was restricted. In conclusion, the effect of the spin-speed of the AR-N 4340 was summarized in Table 1. The width of the top photoresist after developing can be adjusted from 200 nm to 350 nm using a single mold. Changing the spin speed of AR-N 4340 is a good method to adjust the linewidth for large-scale areas (from 200 nm to 350 nm).

It should be noted that the concentration of developer, composing of Tetramethylammonium hydroxide (TMAH) in aqueous solution, was varied according to the NHD. When the NHD was 50 nm, the concentration was as low as 1.2% for better control the process of developing, because it would take a few tens of seconds to expose the substrate. However, when the NHD was 90 nm and 100 nm, dissolving the residual of the top photoresist with 1.2% TMAH would take several minutes, causing a significant developing difference (Figure S7). The concentration of TMAH should be improved to 1.6% when the NHD was 90 nm and 100 nm. It was beneficial to adjust the developing time in few tens of seconds to realize uniform nanostructure and good control of developing process simultaneously. After dissolving the residual of top photoresist, the colour of the developer changes from transparent to yellow, which is caused by dissolving of LOL 2000. This phenomenon can be used to estimate whether the concentration of TMAH was adequate.

### 3.5. Gold Nanohole and Nanoparticle

**Figure 7:**
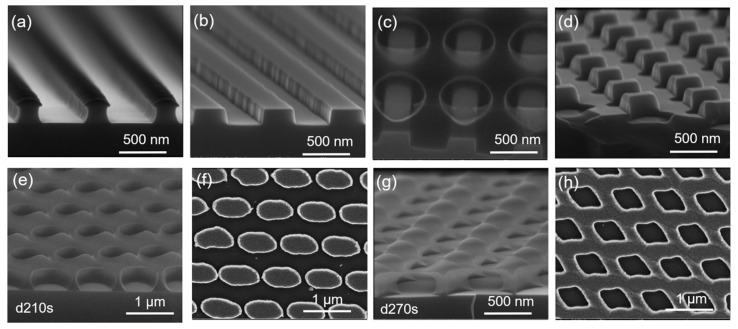
The SEM images during the process of fabricating the gold nanoparticle and nanohole arrays. (**a**) The SEM image of the photoresist after nanoimprinting for the first time. (**b**) The SEM image of silicon gratings after the etching of (**a**). (**c**) The SEM image of the photoresist after nanoimprinting on silicon gratings, rotated by 60°, for the second time. (**d**) The SEM image of the nanoparticle arrays on silicon after the etching of (**c**). Using the PDMS mold replicated from (**d**) to repeat the nanoimprinting process with a developing time of 210 s to obtain the nanoholes presented in (**e**), and with a developing time of 270 s to obtain the nanoparticles shown in (**g**). (**f**) The SEM image of gold nanoparticles after implementing gold evaporation and lift-off on (**e**). (**h**) The SEM image of gold nanoholes after implementing gold evaporation and lift-off on (**g**).

It has been proved that the self-priming nanoimprint technology can be applied on different substrates. Moreover, the double-layer self-priming nanoimprint technology can be extended to fabricate other periodic nanostructures. The nanostructures were expanded to nanoparticle arrays by nanoimprinting twice using the 1D gratings mold via the process depicted in Scheme 2 and the corresponding SEM image in Figure 7. First, the gratings of photoresist on silicon were produced using the double-layer self-priming nanoimprint method, shown in Figure 7a. The exposed silicon was etched by plasma to transfer the gratings to silicon, as demonstrated in Figure 7b. The silicon gratings were coated with a double layer of photoresist and were nanoimprinted through self-priming nanoimprint again by covering PDMS gratings with a rotation angle for the silicon gratings. The SEM image of the photoresist after development is shown in Figure 7c. After etching, the exposed silicon was etched by plasma for the same time again, and the particles on silicon was fabricated (Figure 7d). By pouring the PDMS on the silicon nanoparticle, negative patterns of the silicon particles were formed on PDMS after curing. The AFM images of silicon nanodots and PDMS nanoholes were shown in Figure S4a,b, respectively. The nanohole pattern negative to the nanoparticles on silicon was formed on PDMS. Then, the silicon substrate with a size of 2 cm × 2 cm was nanoimprinted with the PDMS nanohole stamp using double-layer self-priming nanoimprint method. When developing time was 210 s, the photoresist in the centre, surrounded by raised nanoparticles was dissolved to form nanoholes in Figure 7e. When developing time was 270 s, the photoresist in the raised nanoparticles was left to form nanoparticles in Figure 7g. This is another evidence that show the potential of double-layer system in adjusting the parameters of nanostructure by developing. After gold evaporation and lift-off, the gold nanoparticle and nanohole arrays were formed as shown in Figure 7f,h, respectively. It should be noted that the initial mold was 1D circle gratings, therefore, the angle formed by two nanoimprints was changed in a large area, which breaks the uniformity. However, it was still one way to expand the nanostructure of mold by nanoimprinting twice or even thrice. Until now, the gold gratings, nanoholes and nanoparticles have been successfully fabricated using the double-layer self-priming nanoimprint method using DVD as original mold. However, there are some limitations: (1) the double-layer self-priming nanoimprint can perform well in fabricating periodic structures. Nevertheless, if the features of mold are ranging from micrometres to nanometres, the developing difference will appear. (2) Some other complex nanostructures cannot be fabricated using a cheap DVD mold, which would inevitably increase the cost of the master. However, the presented double-layer self-priming nanoimprint method offers the possibility to fabricated nanostructures with different line width and height. Therefore, it is a cost- effective technique cost to adjust the dimensional parameters of the nanopattern using a single mold.

The simple and cheap self-priming nanoimprint technology could be widely applied to fabricate metal nanostructures on different substrates by different kinds of periodic molds with controllable height and width without the assist of expensive instruments. Comparing with other kinds of nanoimprint methods summarized in Table 2, the advantages were list below: (1) no pressure and heating were applied in the process of imprinting, which could get rid of expensive equipment; (2) the curing process after imprint was realized by heating for 150 s, which was time-consuming for high throughput; (3) the evaporated metal can be lifted-off easily by double-layer system, which was much cheaper and easier than dry etch of metal under the photoresist by expensive equipment; (4) the initial mold was commercial DVD, which is cheap and easy to obtain. However, the resolution of this work is 165 nm (Figure 3c) which is lower than the linewidth of mold, it is hard to fabricate structure with a feature size of 10 nm by double-layer self-priming nanoimprint with developing. However, 10 nm resolution can be achieved by combining the double-layer self-priming nanoimprint method with self-aligned double patterning (SADP) [19], atomic layer deposition [32] and controllable metal deposition, including oblique deposition and shadow deposition [33].

### 3.6. Refractive Index Sensor 

One promising application of nanostructured metal is it implementation as a plasmonic sensor. The surface plasmonic resonance (SPR) could be excited by metal nano-gratings. The resonant wavelength is very sensitive to the refractive index of the medium on the metal surface with the electric field enhancement. This property had been widely used in label-free sensing, SERS, and surface-enhanced infrared absorption [36,37,38,39]. The refractive index sensitivity was an important parameter for the plasmonic sensor. The sensitivity in terms of the wavelength shift per refractive index unit (RIU) was determined by [37]

(3) $$S=\Delta \lambda_{res}/\Delta n$$

where *λ_res_* is the resonant wavelength and *n* is the refractive index of the medium on the metal surface. 

**Figure 8:**
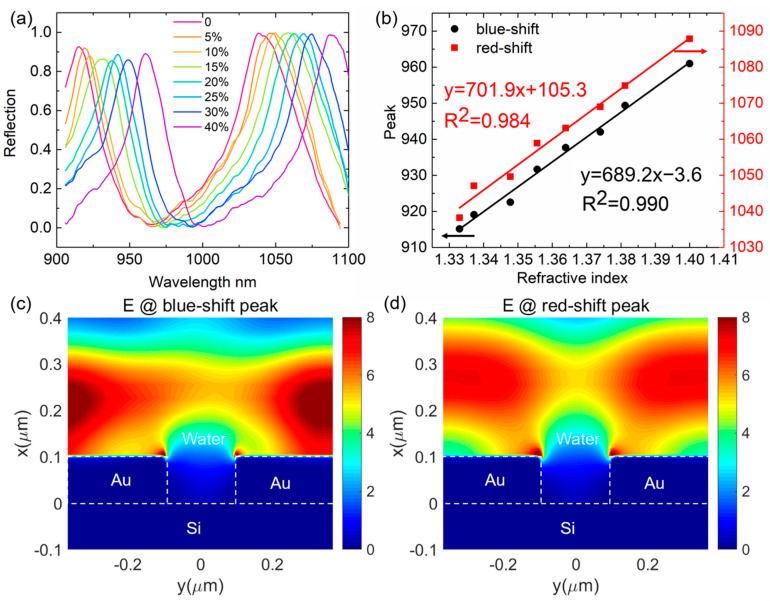
The refractive sensitivity of gold gratings on silicon. (**a**) The reflection spectra of gold gratings immersed in different concentrations of sucrose in water. (**b**) The linear fitting of the blue-shift and red-shift peak position to refractive index. The electric field enhancement distribution of blue-shift and red-shift peak is shown in (**c**,**d**), respectively.

To experimentally demonstrate the sensitivity, the reflection spectra of gold gratings 4 × 4 mm silicon surface were measured by immersing the chip in different concentrations of sucrose aqueous solutions (Figure 8). The concentrations of the sucrose aqueous solutions were 0, 5, 10, 15, 20, 25, 30, and 40 wt %, and the corresponding refractive indexes were 1.3330, 1.3372, 1.3478, 1.3556, 1.3639, 1.3740, 1.3812, and 1.4000, respectively [40]. For an incident angle of 5°, the first order of SPP on gold and medium was split into two branches. By fitting the reflection peaks linearly with the refractive index of sucrose aqueous, the slope of the linear fit indicated the sensitivity of the sensing, which was 701.9 nm/RIU and 689.2 nm/RIU for the red-shift branch and blue-shift peak, respectively. The reflection peaks were both sensitive to the surrounding media owing to the electric field enhancement on the surface of metal and media as shown in Figure 8c,d. The electric field was obtained using Finite-difference time-domain (FDTD) simulation. The sensitivity was larger than that of gold gratings fabricated by FIB with comparable slit width [37,41]. This suggested that the gold gratings fabricated using the cheap double-layer self-priming lithography may attain the same effect as the gold gratings fabricated by FIB for refractive index sensing. The application of this simple and cheap method might open its application in nano-plasmonic biosensing of metal nanostructures.

### 3.7. MoS_2_ Nanogratings

**Figure 9:**
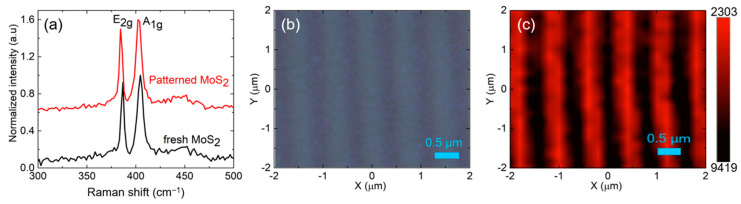
Raman spectra of MoS_2_ nanograting. (**a**) Raman shift of MoS_2_ on an SiO_2_ substrate before and after patterning. (**b**) A microphotograph of an MoS_2_ nanograting on an SiO_2_ substrate. (**c**) Raman shift mapping of the MoS_2_ pattern shown in (**b**) on the SiO_2_ substrate.

The MoS_2_ nanograting on an SiO_2_ substrate fabricated by double-layer self-priming nanoimprint is characterized in Figure 9. As shown in Figure 9a, the Raman spectra shows strong peaks from both the in-plane E_2g_ vibration at 384.7 cm^−1^ and out-of-plane A_1g_ vibration at 403.6 cm^−1^. The frequency difference of 18.9 cm^−1^ between the A_1g_ and E_2g_ modes is a clear signature of the single layer [42,43]. In the microphotograph of the MoS_2_ nanograting on an SiO_2_ substrate (Figure 9b), the brighter part is MoS_2_, and the darker part is SiO_2_. By implementing Raman mapping of the same part in Figure 9b, the nanograting structure can be clearly constructed using the Raman intensity difference, as shown in Figure 9c. The MoS_2_ patterned with the nanostructure can be widely applied to field-effect transistors [43,44], high-Q optical resonators [45], sensing [46] and optoelectronic devices [47]. The Raman characterization proves that the double-layer self-priming nanoimprint method can be applied to pattern 2D materials, such as MoS_2_. Here, we take gold gratings and MoS_2_ gratings as two typical examples to demonstrate the wide application of the nanopatterning of different materials using the double-layer self-priming nanoimprint method.

## 4. Conclusions

In summary, we demonstrated a programmable and cost-effective method to fabricate a nanostructure uniformly on the wafer-scale by implementing a double-layer self-priming nanoimprint method using periodic molds, without the assistance of expensive instruments. No pressure was applied to the PDMS mold and the substrate with the photoresist because the liquid photoresist could automatically fill the nanostructure using capillary force. Furthermore, the double-layer photoresist method is a cheap and simple method to remove the residual photoresist using developing and transfer the nanostructure to the metal using the lift-off process. All processes could be conducted in a chemical solution without expensive laboratory apparatuses. The maximal height of the metal was dependent on the thickness of the bottom photoresist, which can be larger than the height of mold. It provides a programmable route to adjust the thickness of metal using a single stamp. Moreover, the width of the metal can be adjusted by changing the thickness of the top photoresist in a large range (150 nm) and the developing time in a small range (60 nm). Using the DVD as the initial mold, we could successfully fabricate 1D gold gratings on hard and soft substrates. The gratings on a 4-inch silicon wafer were uniform, exhibiting a difference of no more than 30 nm, characterized by the SEM and reflection spectra at different points. Moreover, the peak position in the mm^2^ range was consistent with the difference of 0.3 nm (smaller than the resolution of the spectrograph), which is suitable for a sensor without the influence of a nanostructure difference. The gold gratings can be applied as a sensor with a sensitivity of approximately 700 nm/RIU. Furthermore, silicon nanoparticle arrays were fabricated by combining nanoimprint twice with dry etch. Using the silicon nanoparticle stamp, the gold nanoparticle and nanohole arrays were fabricated using double-layer self-priming nanoimprinting for different developing times. This illustrates the double-layer system can adjust the nanostructure easily by developing using a single mold. Moreover, we also fabricated a 2D material nanoribbon by combining the double-layer self-priming nanoimprint method with plasma etching. Different materials with different nanostructure can be fabricated on hard and soft substrate, which show wide application of double-layer self-priming nanoimprint. All the fabrication process can be conducted without expensive instruments and the parameters of nanostructure can be adjusted by double-layer system using single mold. These reduce the cost of instruments and molds.

## Figures and Tables

**Table 1:** The effect of the spin-speed of top photoresist.

The spin-speed of AR-N-4340	6000 rpm	3000 rpm	2000 rpm
The thickness of AR-N-4340	62 nm	80 nm	105 nm
The nanoimprint height difference (NHD) before development	50 nm	90 nm	100 nm
The width of top photoresist (*w*_1_) after development	200 nm	300 nm	350 nm
Concentration of tetramethylammonium hydroxide (TMAH)	1.2%	1.6%	1.6%

**Table 2:** Comparison of self-priming nanoimprint with other nanoimprint methods.

Imprint Material	Imprint Condition	Curing Process	Transfer Structure	Structure	Resolution	Reference
AR-N-4340	Self-priming	heat	lift-off	grating, particle, hole	165 nm	this work
PS/Polymethyl methacrylate (PMMA) blend film	4 kPa; 180 °C	cool	no	grating	400 nm	Ref. [15]
PC sheet	50 bar; 160 °C	cool	Evaporation without lift-off	nanocone	100 nm	Ref. [34]
ultraviolet (UV)-curable resin	With pressure	UV-curing	template stripping	grating	100 nm	Ref. [35]
UV resist	no	UV-curing	dry etch	nanodot	60 nm	Ref. [17]
poly(methyl methacrylate)	300 psi; 140 °C	cool	no	grating	13 nm	Ref. [19]

## Supplementary Materials

The following are available online at https://www.mdpi.com/2072-666X/12/2/121/s1, Figure S1: The AFM image of 1-D grating mold, Figure S2: The process of development under different developing times with concentration of TMAH 1.6% and the spin speed of LOL and AR-N 4340 both 2000 rpm, Figure S3: The schematic to fabricate gold grating on soft substate (PI and Parylene), Figure S4: The AFM image of nanoparticle mold, Figure S5: The microscope picture of AR-N-4340 patterned by photolithography with different cross-linking temperature, Figure S6: The SEM image of photoresist before development under different imprint time with spin-speed of 6000 rpm, Figure S7: The SEM image of photoresist developed with different concentrations of TMAH.
