# Supplementary material for: Route to Cost-Effective Fabrication of Wafer-Scale Nanostructure through Self-Priming Nanoimprint
Source: Micromachines (Basel). 2021 Jan 24;12(2):121. doi: 10.3390/mi12020121 (PMC7911382; doi:10.3390/mi12020121)
Supplement: Supplementary file 1 [file micromachines-12-00121-s001.pdf]

Supplementary File

Route to cost-effective fabrication of wafer-scale nanostructure through self-priming nanoimprint

Yue Su, Zhaoxin Geng, Weihao Fang, Xiaoqing Lv, Shicai Wang, Zhengtai Ma and Weihua Pei

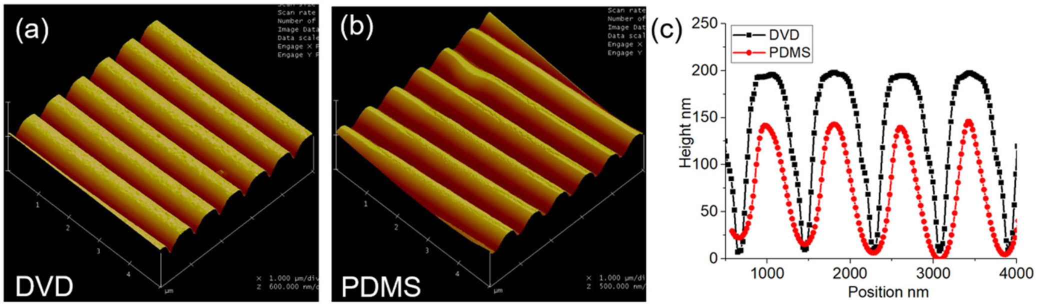

Figure S1. The AFM image of 1-D grating mould. (a) The AFM image of gratings on transparent PC of DVD. (b) The AFM image of PDMS replicated from (a) with negative patterns. (c) Cross-sectional profiles of initial DVD mould and replicated PDMS mould by reversing the profile of PDMS.

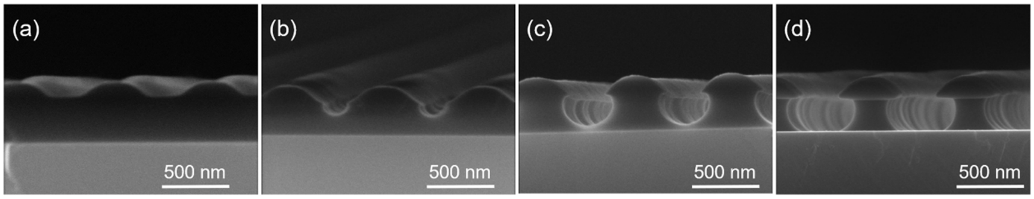

Figure S2. The process of development under different developing times with concentration of TMAH 1.6% and the spin speed of LOL and AR-N 4340 both 2000 rpm. (a) The SEM image of photoresist after developing for 0 s. (b) The SEM image of photoresist after developing for 29 s. (c) The SEM image of photoresist after developing for 37 s. (d) The SEM image of photoresist after developing for 50 s.

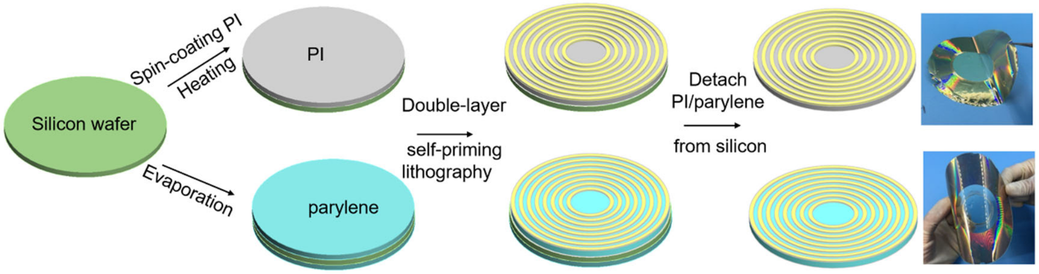

Figure S3. The schematic to fabricate gold grating on soft substate (PI and Parylene).

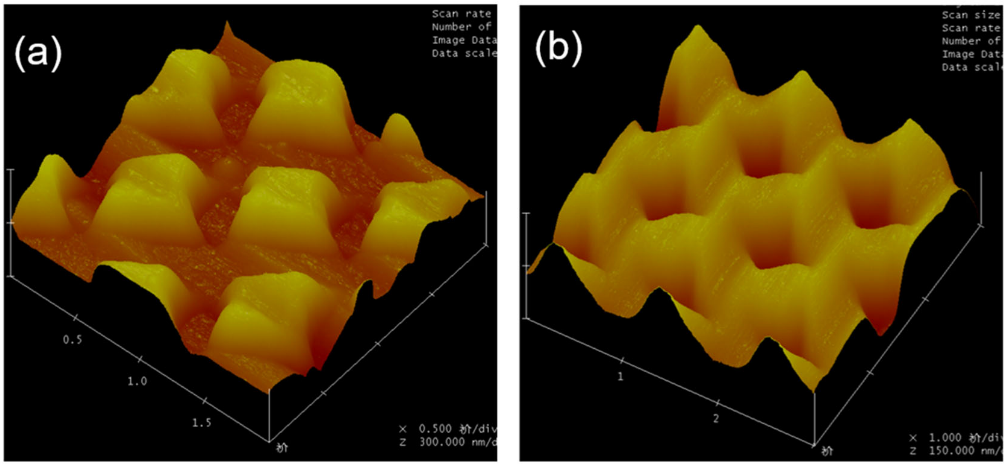

Figure S4. The AFM image of nanoparticle mould. (a) The AFM image of silicon nanopaticles mould produced by two nanoimprints and etching. (b) The AFM image of PDMS replicated from (a) with negative patterns.

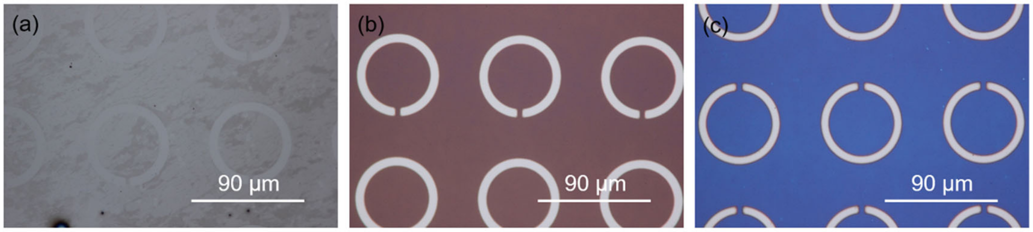

Figure S5. The microscope picture of AR-N-4340 patterned by photolithography with different cross-linking temperature ((a) 100°C; (b) 120°C; (c) 140°C) after developing in 1.6% TMAH for 40 s.

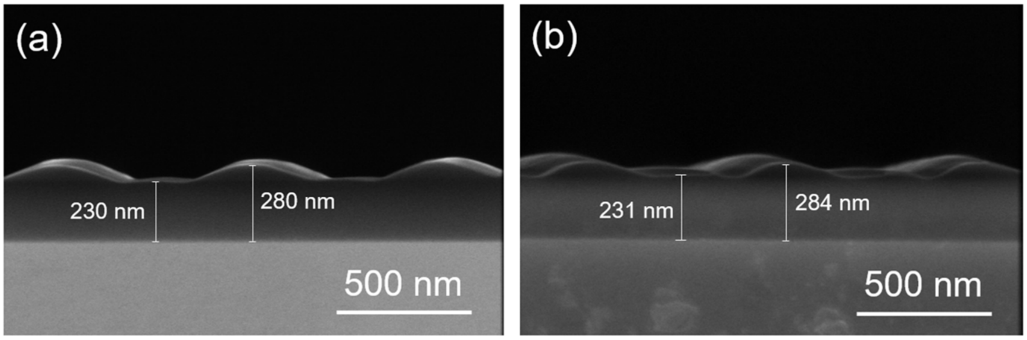

Figure S6. The SEM image of photoresist before development under different imprint time with spin-speed of 6000 rpm. (a) PDMS cover liquid AR-N 4340 for 1 h. (b) PDMS cover liquid AR-N 4340 for 5 min.

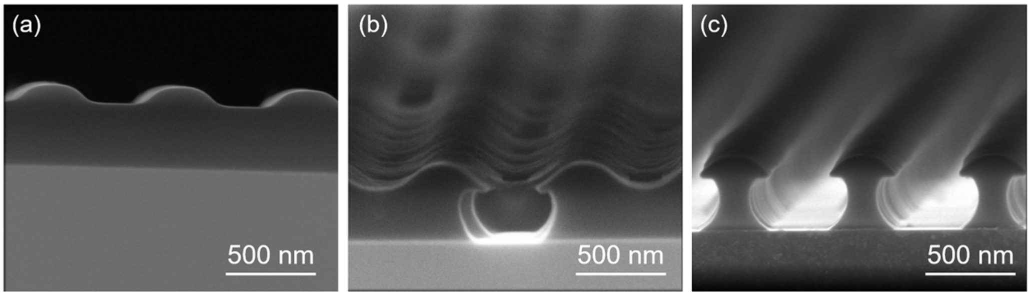

Figure S7. The SEM image of photoresist developed with different concentrations of TMAH. (a) The SEM image before developing with the spin-speed of the top photoresist of 2000 rpm and the bottom photoresist of 2000 rpm. (b) The SEM image of photoresist after developing with TMAH at a concentration of 1.2% for 4 minutes. (c) The SEM image of photoresist after developing with TMAH at the concentration of 1.6% for 40 s.
